# Genome-Wide Characterization, Evolutionary Analysis of *ARF* Gene Family, and the Role of *SaARF4* in Cd Accumulation of *Sedum alfredii* Hance

**DOI:** 10.3390/plants11091273

**Published:** 2022-05-09

**Authors:** Dong Xu, Chunyu Yang, Huijin Fan, Wenmin Qiu, Biyun Huang, Renying Zhuo, Zhengquan He, Haiying Li, Xiaojiao Han

**Affiliations:** 1 Key Laboratory of Three Gorges Regional Plant Genetic & Germplasm Enhancement (CTGU), Biotechnology Research Center, China Three Gorges University, Yichang 443000, China; xudong@caas.cn (D.X.); yangcy1995@foxmail.com (C.Y.); 2 State Key Laboratory of Tree Genetics and Breeding, Chinese Academy of Forestry, Beijing 100091, China; fhj1201@163.com (H.F.); qiuwm05@163.com (W.Q.); hby948750582@163.com (B.H.); zhuory@gmail.com (R.Z.); 3 Key Laboratory of Tree Breeding of Zhejiang Province, The Research Institute of Subtropical of Forestry, Chinese Academy of Forestry, Hangzhou 311400, China; 4 Institute of Virology and Biotechnology, Zhejiang Academy of Agricultural Sciences, Hangzhou 310021, China

**Keywords:** *Sedum alfredii* Hance, *ARF*, collinearity analysis, SNP, cadmium

## Abstract

Auxin response factors (*ARFs*) play important roles in plant development and environmental adaption. However, the function of *ARFs* in cadmium (Cd) accumulation are still unknown. Here, 23 *SaARFs* were detected in the genome of hyperaccumulating ecotype of *Sedum alfredii* Hance (HE), and they were not evenly distributed on the chromosomes. Their protein domains remained highly conservative. *SaARFs* in the phylogenetic tree can be divided into three groups. Genes in the group Ⅰ contained three introns at most. However, over ten introns were found in other two groups. Collinearity relationships were exhibited among ten *SaARFs*. The reasons for generating *SaARFs* may be segmental duplication and rearrangements. Collinearity analysis among different species revealed that more collinear genes of *SaARFs* can be found in the species with close relationships of HE. A total of eight elements in *SaARFs* promoters were related with abiotic stress. The qRT-PCR results indicated that four *SaARFs* can respond to Cd stress. Moreover, that there may be functional redundancy among six *SaARFs*. The adaptive selection and functional divergence analysis indicated that *SaARF4* may undergo positive selection pressure and an adaptive-evolution process. Overexpressing *SaARF4* effectively declined Cd accumulation. Eleven single nucleotide polymorphism (SNP) sites relevant to Cd accumulation can be detected in *SaARF4*. Among them, only one SNP site can alter the sequence of the *SaARF4* protein, but the *SaARF4* mutant of this site did not cause a significant difference in cadmium content, compared with wild-type plants. *SaARFs* may be involved in Cd-stress responses, and *SaARF4* may be applied for decreasing Cd accumulation of plants.

## 1. Introduction

Cadmium (Cd) is released into the environment mainly by industrial activities [1]. This released Cd can enter the human body via the food chain cycle [2], and excess Cd will lead to serious health problems [3]. For crop production, we should decline the Cd content in edible parts of plants to minimize Cd intake for people [2]. However, plants applied in phytoremediation should have the ability to accumulate higher Cd on their aerial parts, as this ability can enhance the effect of phytoremediation for removing Cd from the environment [4]. Whereas plants cannot accumulate excess Cd, as Cd is also toxic to plants [5]. Meanwhile, plants do not effectively separate Cd and essential elements, and Cd is thus inevitably accumulated in edible parts of plants. For example, OsHMA2 can transport zinc and Cd simultaneously [6]. Therefore, it is a requirement for people to flexibly modulate the current status of Cd accumulation in plants according to their different purposes. The transgenic technology is an important strategy to alter Cd accumulation in plants. Furthermore, exploring ideal genes is a precondition for this technology. For example, overexpression of *HMA3* from low Cd-accumulating cultivars of rice can block the Cd translocation process from the roots to above-ground tissues [7]. *SaMT2* from *Sedum alfredii* Hance can significantly increase Cd accumulation of the transgenic tobacco [8]. Therefore, genes related with Cd accumulation in plants may all have the practical application value. Exploring the related novel genes is significant for ensuring food security and controlling Cd pollution.

Up to date, many genes were identified to be relevant with Cd accumulation [9,10]. The functions of these genes can mainly be divided into three groups: The first group is responsible for the intracellular transport of Cd between different organelles. For example, *SpHMA1* protected the photochemical reactions by exporting Cd from chloroplasts to cytoplasm [11]. The second group plays roles in the transmembrane transport of Cd. For example, expressing *OsABCG36* of rice in yeast exhibited efflux activities for Cd [12]. Genes in the third group act on Cd long-distance transportation. For example, *AtHMA4* plays roles in Cd loading in the xylem, and this process was responsible for transporting Cd from the roots to the shoot [13]. These genes associated with Cd accumulation are mainly from *ZIP*, *CDF*, *NRAMPs*, *HMAs*, and *ABC* gene families [14,15,16,17,18]. However, most of them are metal transporters; whether transcription factors could change Cd accumulation remains further confirmed.

Auxin is the initial signal for triggering the expression of downstream genes in the auxin pathway [19]. Moreover, previous studies indicated that auxin can change Cd accumulation [20]. Therefore, genes in auxin pathway may play roles in modifying Cd metabolism in plants. Auxin response factors (*ARFs*) are important transcription factors for the transduction of auxin signals in the auxin pathway [21]. They are involved in almost all the auxin-related processes [22]. For example, overexpression of *ARF4* affected the salt resistance of poplars by inhibiting the lateral root development [23]. Decreasing the expression of *SlMIR160a* and *SlARF10A* in tomato can restore the abnormal phenotypes of leaves and flowers of tomato *CR-slmir160a* mutants [24]. However, it is unclear that *ARFs* are responsible for the Cd accumulation process.

The hyperaccumulating ecotype of S. *Alfredii* (HE) is one of the most famous Cd hyperaccumulators [25,26]. Recently, it has been applied for harnessing Cd-contaminated soils [27]. Although HE has an excellent ability of accumulating Cd in aerial parts of plants, another ecotype of *S. alfredii* (NHE) is unable to accumulate excessive Cd [28]. Therefore, the two ecotypes have been become the ideal materials for studying the Cd accumulation mechanisms [29,30]. For example, *HMA3* from HE was transformed into NHE to identify its roles in the Cd accumulation process [16]. However, the lack of genomic information of HE blocked the identification of candidate genes. We finished the whole-genome sequencing of HE and NHE (unpublished), which can be helpful for the genome-wide detection and analysis of candidate genes in the past few years.

In this study, we detected 23 ARF genes from the HE genome. The candidate genes with their possible roles in Cd accumulation were screened from the *ARF* gene family, mainly based on the evolutionary analysis. Then, the transgenic method was used for verifying the roles of the candidate genes in Cd accumulation, and this function was further demonstrated by generating the key single nucleotide polymorphism (SNP) mutant of the candidate gene.

## 2. Results

### 2.1. Phylogenetic Analysis, Protein Domains, Promoter Motifs, Exons, and Introns of SaARFs

**Figure 1:**
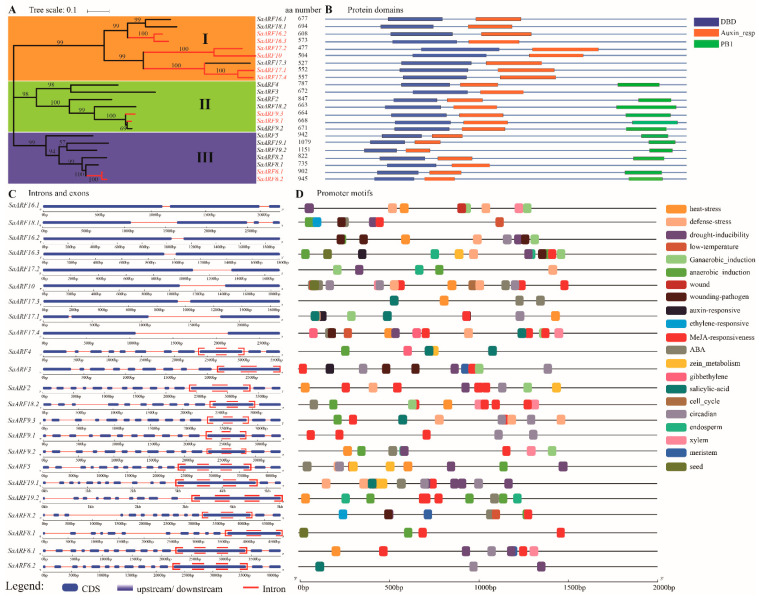
Phylogenetic analysis and sequence analysis of *SaARFs.* (**A**) Phylogenetic tree and amino acid number for each gene. Members signed by red were collinearity genes. aa, amino acid. (**B**) Protein domains, different colors indicated different domains. The blue, orange, and green areas indicated the DBD, Auxin_resp and PB1 domain, respectively. (**C**) Exons (blue areas) and introns (red lines). (**D**) Promoter motifs. The motifs were displayed, which were related with plant hormones, abiotic stress, and plant development. Different colors indicated different motifs.

A total of 23 *SaARFs* were detected in the HE genome (Supplementary Material S5). The basic information of *SaARF* proteins is listed in Table S2, including their genomic location, molecular weight, protein isoelectric point, extinction coefficient, length, and hydrophilicity. The phylogenetic tree of the *SaARF* gene family was constructed, and three clades were thus divided (Figure 1A). The protein domains, the DNA binding domain (DBD), and the auxin_responsive domain (Auxin_resp), can be detected in all *SaARFs*. The first clade contained nine members, and these members all lacked the Phox and Bem1 (PB1) domain (Figure 1B). Conversely, the PB1 domain can be found in other members (except *SaARF3* and *SaARF8*). Then, we analyzed the composition of the introns and exons of these *SaARFs*. The results showed that many introns (at least 10 introns) can be observed in members of the second and third two clades (Figure 1C, the red lines). Furthermore, the longest exon (full or at least part of its sequences) in each gene was located in the PB1 domain (Figure 1C, the red dotted boxes). In sharp contrast, very few introns (at most three introns) were contained in members of the first clade. For example, *SaARF18* in the first clade only contained three introns in its full-length sequences. After that, the promoter–element analysis of *SaARFs* revealed that the elements related with abiotic stress responses, developmental processes, and plant hormones can be detected in their promoters (Figure 1D).

### 2.2. Genomic Location and Collinearity Analysis of SaARFs

**Figure 2:**
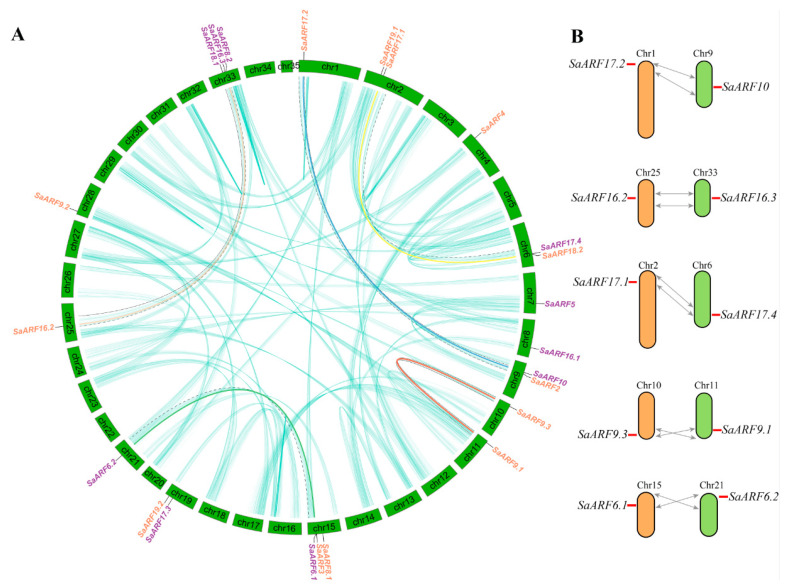
Genomic localization and hypothetical evolutionary histories of *SaARFs.* (**A**) The distribution of *SaARFs* in chromosomes and the collinearity relationships throughout the whole genome. The cyan lines indicated the collinearity relationships of genes (except *SaARFs*). Other colored lines indicated the collinearity relationships among *SaARFs*. The genes marked by purple IDs were located in the positive strand of DNA, while others signed by orange IDs were in negative strand. The black dotted lines indicated the collinearity area. (**B**) The grey lines indicated the predicted modes of chromosomal segment duplication.

Analyzing the distribution of *ARFs* in chromosomes may contribute to clarify the evolutionary history of the gene family [31]. We found that *SaARFs* were uneven throughout chromosomes. In detail (Figure 2A), only one member was each situated on chromosomes 1, 4, 7, 8, 10, 11, 21, 25, 28; two *SaARFs* positioned each in chromosomes 2, 6, 9, 19; while chromosome 15 and 33 each contained three *SaARFs*. Whereas no *SaARFs* were located in other chromosomes.

The segmental and tandem gene duplication are considered as the two important reasons for generating gene family members [32]. The collinearity relationships across the whole HE genome were detected to identify the reasons for *SaARFs* formation. The results showed that there were collinearity relationships between five pairs of *SaARF* members (Figure 2A,B). Meanwhile, most of them were distributed in the first clade of the phylogenetic tree (Figure 1A, red lines). We also found that these genes were all located in the corresponding collinear blocks of the genome (Figure 2A, indicated by black dotted lines). Combined with the possible reasons for the formation of these blocks (Figure 2B, the grey arrows), we considered that the segmental duplication and rearrangements may be two important reasons for the generation of these *SaARFs* (Figure 2B).

### 2.3. Comparative Genome Analysis of ARFs among Different Species

**Figure 3:**
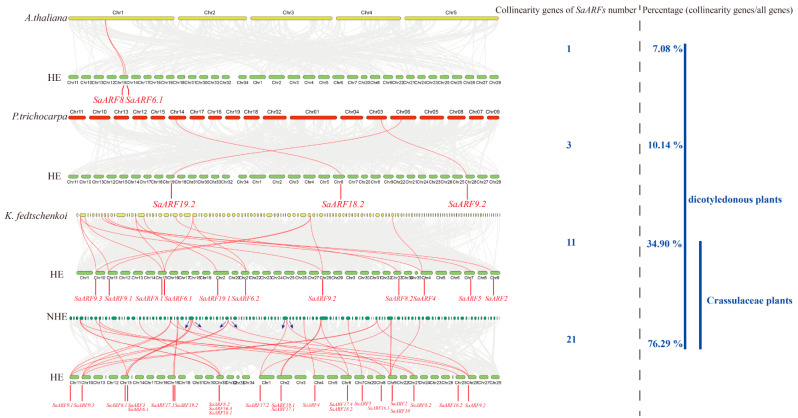
Collinearity relationships between HE and other species. The grey lines indicated collinearity relationships of other genes (except *SaARFs*). The red lines indicated the collinearity relationships of *SaARFs*. The blue arrows indicated that one *ARF* gene of NHE corresponded to two or three *ARF* genes of HE.

Collinearity relationships usually exist between genes originated from common ancestors in angiosperms [33]. As HE is a dicotyledonous plant, we analyzed the collinearity relationships of HE genes with those in two dicotyledonous plants (*Arabidopsis thaliana* and *Populus trichocarpa*) and two *Crassulaceae* plants (*Kalanchoe fedtschenkoi* and NHE) to clarify the phylogenetic mechanisms of *ARF* family. Our results indicated that 7.08% of *A. thaliana* genes have collinearity relationships with genes in HE, while this percentage was 10.14% in *P. trichocarpa*. In contrast, it is much higher in the corresponding percentages in *K. fedtschenkoi* and NHE (34.90% and 76.29%, respectively) than those in *A. thaliana* and *P. trichocarpa* (Figure 3). In other words, the higher percentage can be observed in the species that are more closely related to HE. Meanwhile, we found that one gene in *Arabidopsis* and three genes in *P. trichocarpa* have collinearity relationships with *SaARF6.1*, *SaARF8.1* and *SaARF9.2*, *SaARF18.2*, and *SaARF19.2*, respectively. However, a large amount of collinearity genes of *SaARFs* can be found in NHE and *K. fedtschenkoi* genome (21 collinearity genes and 11 collinearity genes, respectively). The corresponding collinearity genes of all 23 *SaARFs* in HE can be detected in NHE genome. Moreover, one collinearity gene in NHE usually corresponded to two or three *SaARFs* in HE (Figure 3, the blue arrows). These results indicated that more collinearity genes of *SaARFs* can be found in the species that have a close relationship with HE.

### 2.4. Expression Patterns of SaARFs under Cd Stress

**Figure 4:**
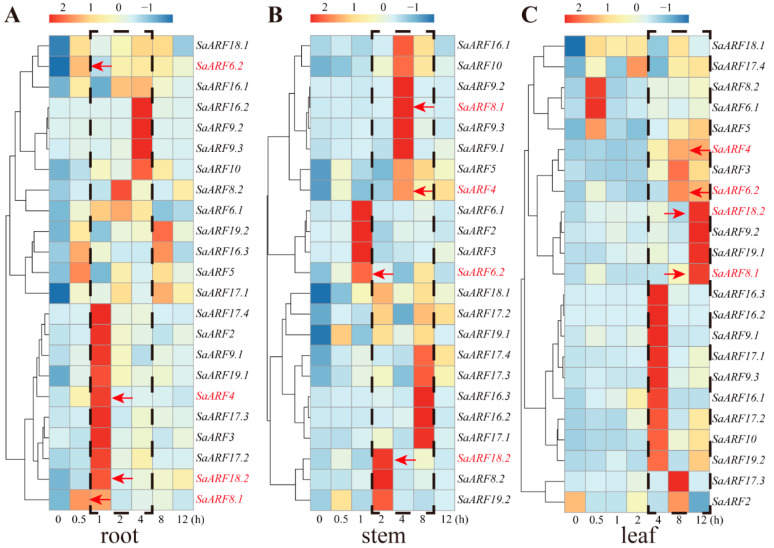
Expression patterns of *SaARFs* in different organs under Cd stress. (**A**)The expression level of 14 *SaARFs* in roots under Cd stress. The black dotted box indicated the highest expression level of 14 *SaARFs* in roots were observed during 1–4 h. The red arrows indicated the highest level of corresponding *SaARFs* during Cd stress. (**B**) The expression level of 18 *SaARFs* in stems under Cd stress. The black dotted box indicated the peak values of 18 SaARFs expressions were distributed between 2–8 h. (**C**) The expression level of 18 *SaARFs* in leaves under Cd stress. The black dotted box indicated the peak values of 18 SaARFs expressions in leaves were mainly found after 4 h.

Then we needed to identify whether the expression of SaARFs are affected by Cd stress. We detected *SaARFs* expression in different organs during 0–12 h under Cd stress. The qRT-PCR results indicated that the expression of *SaARFs* can respond to Cd stress. In general, the highest expression level of 14 *SaARFs* in roots were observed during 1–4 h under Cd stress (Figure 4A, the black dotted box); in stems, the peak values of 18 *SaARFs* expressions were distributed between 2 h and 8 h (Figure 4B, the black dotted box); however, the peak values of 18 *SaARFs* expressions in leaves were mainly found after 4 h under Cd stress (Figure 4C, the black dotted box). In other words, the responses of *SaARFs* in roots seemed to be faster than those in the two other organs, and their responses in leaves were slower than those of other organs. This trend was consistent with the order in the Cd signal delivered in plants. We considered that the response rate of SaARFs under Cd stress should be in accordance with this order. Therefore, *SaARF4*, *SaARF6.2*, *SaARF8.1*, and *SaARF18.2* in 23 *SaARFs* may be the ideal candidate genes (Figure 4). For example, the peak value of *SaARF18.2* expression was observed in the roots at 1 h after Cd treatment (Figure 4A, *SaARF18.2*, the red arrow). However, the expression level of *SaARF18.2* in the stems was highest at the 2 h (Figure 4B, *SaARF18.2*, the red arrow), while the highest expression of *SaARF18.2* was detected in leaves at the 12 h (Figure 4C, *SaARF18.2*, the red arrow).

### 2.5. Coexpression Network Analysis

**Figure 5:**
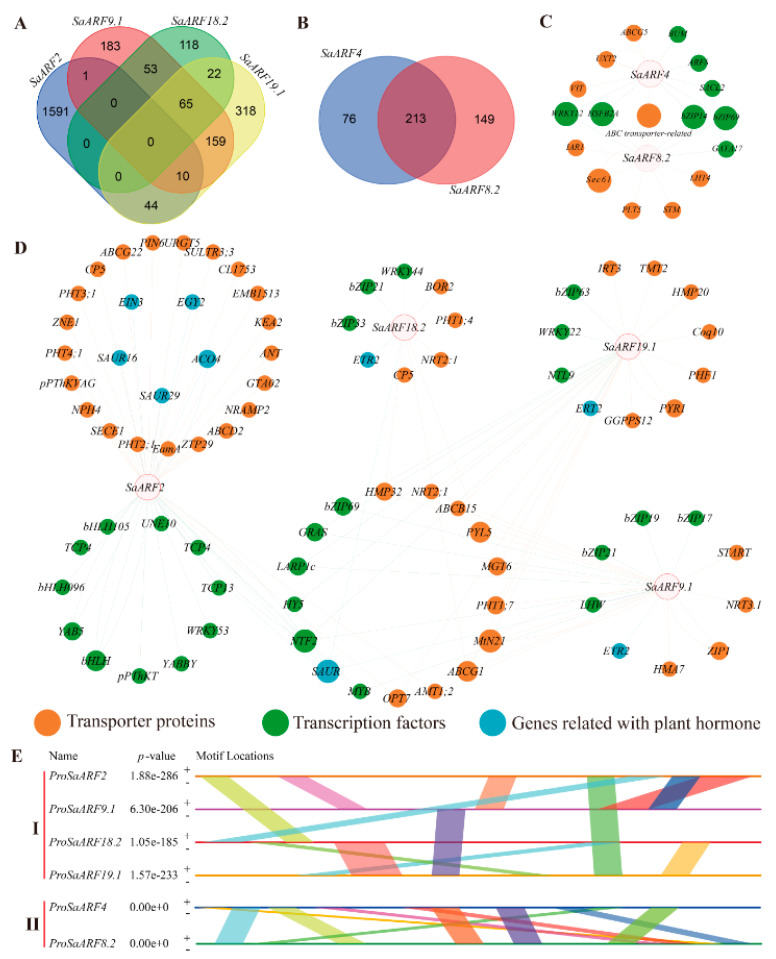
Similar segments of the hub-gene promoters and coexpression network analysis. (**A**) All related genes in the group of *SaARF2*, *9*, *18.2*, and *19*. (**B**) The related genes in the group of *SaARF4* and *8.2*. (**C**,**D**) showed the coexpression network containing the transcription factors, transporter proteins and genes related with plant hormone. (**E**) Similar segments in the promoters of hub genes. The same color in each group indicated a common similar segment.

As the typical transcription factors, *SaARFs* should be controlled by upstream genes, and they can regulate the expression of downstream genes. Therefore, there should be strong expression relationships between *SaARFs* and the related genes. The transcriptome data obtained under Cd stress were used for generating a coexpression network [34], and the related information about *SaARFs* was extracted from the coexpression network. We found that six *SaARFs* (*SaARF2*, *SaARF4*, *SaARF8.2*, *SaARF9*, *SaARF18.2*, and *SaARF19*) in 23 *SaARFs* were identified as hub genes in the coexpression network. The expression of 3002 genes was related with the six *SaARFs* (Figure 5A,B). Moreover, further analysis of these related genes indicated that the six *SaARFs* can be divided into two groups, and there were lots of common genes in each group (Figure 5A,B). Our attention was mainly focused on the transcription factors, transporters, and the genes involved in the pathway of plant hormone. The results indicated that the transcription factors in the coexpression network were mainly distributed in *WRKY*, *bZIP*, and *bHLH* gene families; many ABC transporters were contained in the transporters group; meanwhile, the genes related with the ethylene and auxin pathway were also found in the coexpression network (Figure 5C,D). After that, we extracted the promoter sequences of the hub genes and analyzed the similar segments in these sequences. The results indicated that many similar segments of promoters can be found in each group (Figure 5E), indicating that they may be regulated by the same or similar factors.

### 2.6. Functional Divergence and Positive Selection Analysis

**Figure 6:**
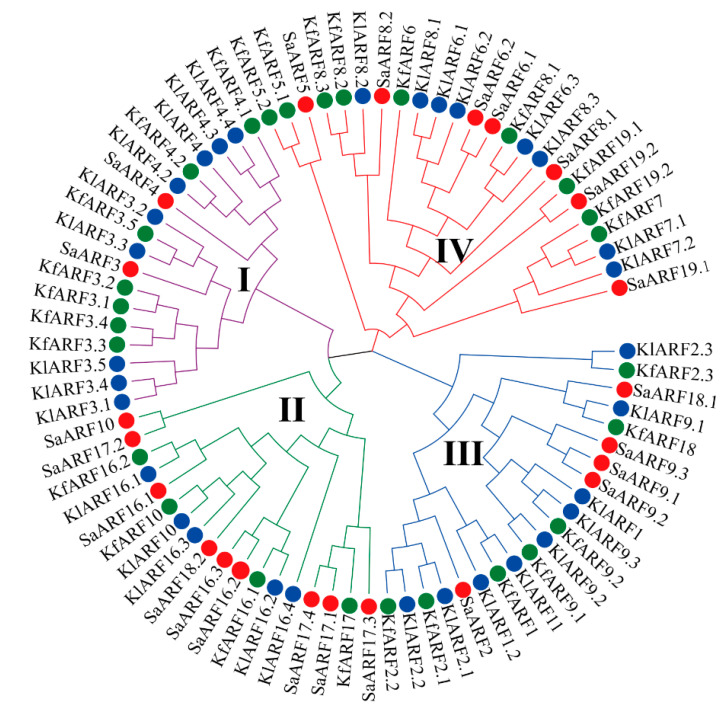
Phylogenetic analysis of ARFs among three *Crassulaceae* plants. I, II, III, IV indicated the first class to the fourth class in the figure. The circles with same color indicated the *ARFs* were in the same species. Green circle, *K. fedtschenkoi*; red circle, HE; blue circle, *K. laxiflora*.

Genes that contain positive selection loci are subjected to selection pressure of adaptive evolution [35]. The paml4.8 was used to identify which *SaARFs* contained the positive selection sites. Two other *Crassulaceae* plants (*K. fedtschenkoi* and *K. laxiflora*) and HE were together chosen for further analysis (Supplementary Material S5). Among them, only HE was the Cd hyperaccumulator plant. A total of 81 *ARFs* were detected, and these *ARFs* can be divided into four clades (Figure 6). We adapted the branch site model for analyzing positive selection sites. The results indicated that 10 positive selection sites (1 M, 9 T, 18 T, 22 I, 30 Q, 36 A, 38 T, 40 E, 42 C, and 43 H) were distributed in the first clade (Table 1). Only two sites were detected in the second clade (6M and 23S), and few sites were in other clades. Furthermore, the functional divergence analysis showed that the first clade has significant functional differentiation compared with other clades (*p* < 0.01, Table 2). Only *SaARF3* and *SaARF4* were distributed into the first clade. In other words, *SaARF3* and *SaARF4* may undergo the positive selection pressure. According to the above analysis, *SaARF4* was chosen for identifying its function in Cd accumulation.

### 2.7. Overexpressing SaARF4 Decreases Cd Accumulation

In order to identify whether the candidate gene, *SaARF4*, could affect Cd accumulation, we transformed *35S::SaARF4* into NHE, and then we measured the Cd accumulation of the overexpression transgenic lines. Furthermore, we performed the whole-genome resequencing to identify the possible SNP sites related with Cd accumulation using more than 100 natural individuals (Supplementary Material S6). We found that 10 SNP sites with *p* < 0.01 were distributed in *SaARF4* sequences (three in upstream sequences and seven in coding-region sequences, Figure S3). Only one SNP can change the protein sequences of *SaARF4*. Thus, we used overlapping-PCR technology to restructure *SaARF4* sequences (*SaARF4-SNPm*). After that, *35S::SaARF4-SNPm* was also transformed into NHE to identify whether this site could influence Cd accumulation.

**Figure 7:**
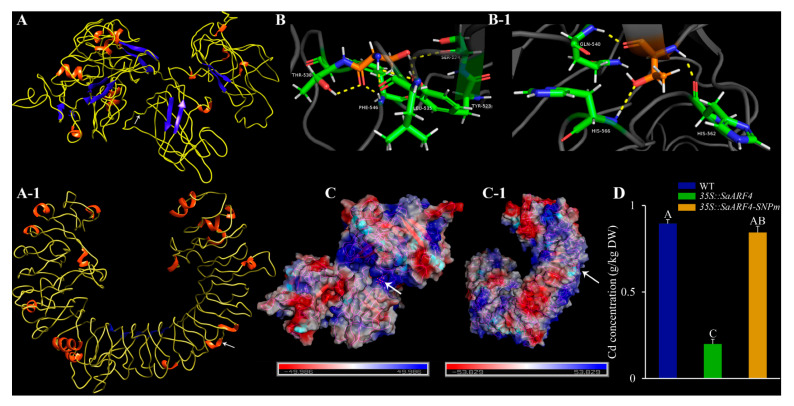
Variations in protein structures of *SaARF4*-SNPm and Cd content of transgenic plants. The protein structures of *SaARF4* and *SaARF4*-SNPm were displayed in (**A**) and (**A-1**), respectively. (**B**) (*SaARF4*) and (**B-1**) (*SaARF4*-SNPm) indicated the mutant aa (the orange one) and its surrounding interaction aa (the green ones). The charge distribution of *SaARF4* and *SaARF4*-SNPm was showed in (**C**) and (**C-1**), respectively. The red color indicated the negative charge; the blue color indicated the positive charge; the white color indicated the neutral charge; the white arrows indicated the positions of the mutant sites. (**D**) The Cd accumulation of NHE WT plants (the blue color), *35S::SaARF4* (the green color) and *35S::SaARF4-SNPm* (the orange color). DW, dry weight. Different capitals indicated different significance, *p* < 0.01.

As shown in Figure 7, the structure of *SaARF4*-SNPm was seriously changed (Figure 7A-1) compared with *SaARF4* (Figure 7A). The interaction proteins surrounding the mutant amino acid (aa) were changed from five aa (TYR-523, SER-524, LEU-535, THR-538, and PHE-516) of *SaARF4* (Figure 7B, the orange one) to three aa (GLN-540, HIS-562, and HIS-566) of *SaARF4*-SNPm (Figure 7B-1, the orange one). Furthermore, the charge distribution of the two proteins indicated that the surrounding charges of the mutant AA (Figure 7C, the white arrow) were altered from the positive charges (Figure 7C, the blue areas) to the neutral charge (Figure 7C-1, the white areas). We also found that overexpressing *SaARF4* can effectively decline the Cd content compared with WT plants of NHE (*p* < 0.01). However, the overexpression of *SaARF4-SNPm* did not significantly decrease Cd accumulation (Figure 7D), indicating that this SNP may play important roles in Cd accumulation.

## 3. Discussion

In this study, a total of 23 *SaARFs* were identified across the whole genome, and then their phylogenetic analysis (Figure 1A), structure analysis (Figure 1B,C), chromosomal location (Figure 2A), and coexpression network analysis (Figure 5) were made. Furthermore, the combination with other species, the collinearity analysis (Figure 3), the positive selection analysis (Figure 6 and Table 1), and the functional divergence analysis (Table 2) were conducted. Meanwhile, qRT-PCR analysis indicated that their expression patterns were altered with different Cd-treatment times (Figure 4). Based on these results, we finally chose *SaARF4* as the candidate gene for further analysis. The result of the transgenic experiment showed that overexpressing *SaARF4* can effectively decrease Cd content (Figure 7D). In order to further determine the effect of *SaARF4* on Cd accumulation, the SNP sites related with Cd accumulation were detected in *SaARF4* sequences. Among these SNP sites, only one SNP can change *SaARF4* protein sequences. We artificially altered *SaARF4* sequences based on the SNP site. The results indicated that overexpression of the mutant sequences did not significantly decrease the Cd content (Figure 7D).

Eukaryotic genes are divided into intron-less (no introns), intron-poor (at most three introns), and intron-rich [36]. A recent study indicated that intron-poor genes were evolved from a subfamily of genes that are intron-rich [37]. We found that 23 *SaARFs* can be divided into three groups, and genes in the first group contained at most three introns (Figure 1A,C). Moreover, the branch length of the first group was longer than those of other groups (Figure 1A). New members of a gene family are produced by gene duplication, and the evolutionary process makes their sequences divergent to produce different proteins. Interestingly, in this study, the collinearity genes in the *ARF* family (Figure 1A, the red lines) were mainly also distributed in the first group, indicating that these genes may be undergoing the evolutionary process. This evidence indicates that the evolution of these genes may be later than other *SaARFs*. Furthermore, the PB1 domain in *ARFs* is responsible for the response to auxin level in plants [21]. In detail, AUX/IAA proteins can bind to the PB1 domain to repress ARFs functions under low auxin concentrations. However, the AUX/IAA proteins will be degraded under a high auxin level, and the *ARFs* are thus released to play their roles on the relevant downstream genes [21]. Therefore, after removing the PB1 domain from *ARFs*, the transformed plants exhibited ‘high auxin’ phenotypes (even though, under low auxin concentration) when overexpressing these *ARFs* mutants [38]. This domain was absent from the genes in the first group (Figure 1B), indicating that the regulatory effect of auxin concentration on their expression may be weaker than other *ARFs*. Furthermore, in the second and third groups, the sequences of the longest exon were all or partly located in the PB1 domain (Figure 1C, the red dotted boxes), indicating that the translation product of this domain may be more stable than other domains with a low effect of differential splicing. This may be required for the fine regulation of *ARFs* by auxin.

Collinearity genes are considered to evolute from the same ancestor [39]. The construction of collinearity relationships throughout the whole genome will be conductive to clarify the evolutionary history of a gene family [40]. In general, the segmental and tandem gene duplication are two important ways for generating new members of a gene family [39]. In this study, we found that there were collinearity relationships between five pairs of *SaARFs* (Figure 2). Meanwhile, they were located in the collinearity blocks of the HE genome (Figure 2A), indicating that the segmental duplication may be a reason for the generation of these *SaARFs*. When comparing the collinearity relationships among different species, the results indicated that the number of collinearity genes of *SaARFs* in *Crassulaceae* plants was more than those in two other species (Figure 3). Moreover, there were positive relationships between the number of collinearity genes of *SaARFs* in different species and the amount of all collinearity genes in different species (Figure 3). In other words, more collinearity genes of *SaARFs* can be detected in the genome of the species that is closely related with HE. In combination with previous studies, we found that *ARFs* seemed to play roles in the nodes of plant differentiation. For example, roots are important for plants to adapt to the terrestrial environment. Correspondingly, *ARF2*, *ARF3*, and *ARF4* are relevant for the development of lateral roots [41]. The developmental differences of flower organs are significant between gymnosperms and angiosperms. *SlARF10A* is critical for flower development [42]. Meanwhile, herbaceous and woody plants are distinguished by the differences in xylem development. In poplars, *PtoARF5* has been demonstrated to be related with the secondary xylem development [43]. Therefore, our results and previous studies together verified that the *ARF* gene family may play important roles in species differentiation.

There was functional redundancy among *SaARFs*, based on the results of the coexpression network (Figure 5). For example, a total of 289 genes have close expression relationships with *SaARF4* (Figure 5B). Among them, the expression of 213 genes was also relevant with SaARF8.2. Furthermore, similar sequences can also be found in their promoters (Figure 5E), indicating that they may be regulated by similar or same factors. This phenomenon can also be found in the *ARF* family of *Arabidopsis*. Okushima reported that *ARF7* and *ARF19* in *Arabidopsis* together act in the lateral root formation, and overlapping functions were between them [44]. According to previous studies, ARFs mainly acted in developmental events of plants [45,46]. Therefore, overlapping functions among *ARFs* may be an important strategy for plants to ensure the normal development, because if one gene was impaired, another can rescue this deficit. In this study, our attention was the influence of the candidate gene on Cd accumulation. The Cd accumulation of transgenic plants was the phenotype that we focused on. The protein structure and surface charge distribution of a transcription factor are important factors to affect its binding with the downstream gene promoter [47,48,49]. We found that the surface charge distribution and protein structure of *SaARF4* were all altered (Figure 7A,A-1,C,C-1), after changing its protein sequences using the method of site-directed mutagenesis. Therefore, the downstream genes regulated by *SaARF4* may be different from those genes controlled by *SaARF4-SNPm*. Interestingly, the Cd accumulation of *35S::SaARF4* was significantly decreased, compared with those of NHE WT plants. However, the significant difference in Cd accumulation was not observed between the *35S::SaARF4-SNPm* and WT plants (Figure 7D). This evidence indicated that *SaARF4* and its downstream genes may play important roles in Cd accumulation. We will explain the detailed mechanism about how *SaARF4* regulates its downstream genes to decrease Cd accumulation in a further study.

## 4. Methods and Materials

### 4.1. Plant Materials

HE and NHE were originally obtained from Quzhou [50] and Fuyang [16] countries, respectively, Zhejiang Province, China. The branches from these materials were used for cuttage propagation under the condition of 25 °C, 16 h light/8 h dark cycle.

### 4.2. Genome-Wide Identification of ARFs in Different Species

Genome sequencing of HE and NHE were performed, and then chromatin assembly of HE was also finished. The protein sequence file of HE was used for screening *ARF* genes. Except for HE and NHE, the genomic files of other species were all downloaded from Phytozome (https://phytozome.jgi.doe.gov/pz/portal.html (accessed on 18 March 2022)) [51]. The Hidden Markov Model (HMM) profile of the Auxin_resp domain (PF06507.12) was downloaded from pfam website (http://pfam.sanger.ac.uk/ (accessed on 18 March 2022)). Then, these protein sequence files and the pfam profile were used for identification of *ARFs* on the platform of SPDE [52]. Genes with default *E*-value (<0.1) were considered as *ARFs*. The intactness of conserved protein domains was tested by using NCBI-BLASTP (https://blast.ncbi.nlm.nih.gov/Blast.cgi (accessed on 18 March 2022)), and the incorrect genes were removed. After obtaining the gene identifications (IDs), SPDE was used for extracting the relevant sequences in batch. After that, these obtained sequences were blasted to the database using the website of TAIR (https://www.arabidopsis.org/ (accessed on 18 March 2022)) and named according to their homologous genes in *Arabidopsis*. If two or more genes have a same homologous gene of *Arabidopsis*, they were named as 1.1, 1.2, etc.

### 4.3. Phylogenetic Analysis, Protein Structures, Promoter Elements, Exons, and Introns

The phylogenetic trees were constructed using MEGA-X (https://www.megasoftware.net/ (accessed on 18 March 2022)) with Minimal Evolution, Neighbor-Joining, and maximum likelihood (ML) methods (bootstrap, 1000). Only ML tree was displayed, as the three trees were consistent. The NCBI-BLASTP (https://blast.ncbi.nlm.nih.gov/Blast.cgi (accessed on 18 March 2022)) was used to identify the protein-domain locations of *SaARFs*. The promoter analysis of *SaARFs* was conducted using PlantCARE (http://bioinformatics.psb.ugent.be/webtools/plantcare/html/ (accessed on 18 March 2022)). Then, GSDS2.0 (http://gsds.gao-lab.org/ (accessed on 18 March 2022)) and TBtools [53] were used for displaying the protein structures, promoter elements, exons, and introns.

### 4.4. Chromosomal Locations and Collinearity Analysis

The required files were generated by SPDE using genomic data. Then, these files were used to display the genomic information by the software of Circos (http://circos.ca/ (accessed on 18 March 2022)). The collinearity analysis was made by MCScanX software with *E*-value < 1 × 10^−5^ (http://chibba.pgml.uga.edu/mcscan2/ (accessed on 18 March 2022)). Other operations were conducted as stated in the manual.

### 4.5. Comparative Genomics Analysis

The related genomic files were downloaded from Phytozome. Then, these files were used for comparing the genomic information between different species with TBtools.

### 4.6. Coexpression Network Construction

The data reported in our previous studies [34] were used for extracting the relevant information to construct the coexpression network. After identification of hub genes in *SaARFs*, the 2000 bp promoter sequences of the hub genes were extracted from genome sequences using SPDE. Then, the conserved segments of these sequences were detected using the website of MEME (http://meme-suite.org/tools/meme (accessed on 18 March 2022)). The venn diagrams were drawn by Bioinfrmatics & Evolutionary Genomics (http://bioinformatics.psb.ugent.be/webtools/Venn/ (accessed on 18 March 2022)). The cytoscape software was used for constructing the coexpression network [54]. After that, the images were further processed by Abode Illustrator.

### 4.7. Functional Divergence and Positive Selection Analysis

The functional divergence of *SaARFs* was analyzed using DIVERGE v3.0 program [55]. The positive selection analysis was conducted by using the PAML package, and the parameters were set as previously described [56].

### 4.8. Quantitative Reverse Transcription-PCR (qRT-PCR)

Wild-type (WT) plants of HE were water-cultured under the condition (25 °C, 16 h light/8 h dark cycle) for about 3 weeks. Then, these materials were treated under 400 μM CdCl_2_. The roots, stems and leaves were collected at 0 h, 0.5 h, 1 h, 2 h, 4 h, 8 h and 12 h, respectively. Each sample with three biological replicates was immediately put into liquid nitrogen for RNA extraction. Total RNA was extracted from leaves using an RNA extraction kit (RNAprep Pure Plant Kit, TIANGEN, Beijing, China) followed by DNase I to remove genomic DNA. Next, first-strand cDNA was produced using the cDNA synthesis kit (PrimeScript™ RT Master Mix, TAKARA, Beijing, China). qRT-PCR was performed as Shuang-Shuang et al. described [50]. The primers were designed by SPDE in batch and listed in the Table S1.

### 4.9. SNP Sites Detection and Three-Dimensional Structure Visualization

After sequencing HE genome, we collected 106 individuals (including 52 HE and 54 NHE) from eleven natural populations. Then, the whole-genome resequencing approach was performed to SNP sites. Meanwhile, we determined the Cd content of the 106 individuals. Association analysis between SNP sites and Cd content was made using Tassel 5 [57], and SNP sites with *p*-value < 0.01 were remained. The SNP site that can change the protein sequence of *SaARF4* was utilized for further functional verification. The three-dimensional structures of proteins were constructed using I-TASSER website (https://zhanglab.ccmb.med.umich.edu/I-TASSER/ (accessed on 18 March 2022)), and then the software of Chimera was used for displaying their three-dimensional structures [58].

### 4.10. Plasmid Construction and Transgenic Experiment

Total RNA was extracted from the HE leaves, according to the above method (stated in Section 4.8). Then, the total RNA (2 µg) was used for synthesizing the first-strand cDNA by Superscript RT III first-strand cDNA synthesis kit (Invitrogen, Carlsbad, NM, USA) followed by RNase H treatment. The primers for *SaARF4* clone were listed in Table S1. The coding sequence of *SaARF4* was cloned from HE cDNA. Furthermore, the sequence mutant of *SaARF4* (*SaARF4-SNPm*) was produced using overlapping PCR, according to the results of SNP analysis. The vectors, pDONR222 and pK2GW7.0, were used for constructing *35S::SaARF4* and *35S::SaARF4-SNPm*, respectively. Next, the recombinant vectors were transformed into the *Agrobacterium tumefeciens* strain EHA105. The NHE leaves were used for *A. tumefeciens* infection. The transgenic experiment was performed as previously described [16]. Minor modifications: differentiation medium: MS + 2 mg·L^−1^ 6-benzylaminopurine + 0.3 mg·L^−1^ 1-naphthaleneacetic acid; rooting medium: 1/2 MS + 2 mg·L^−1^ 3-indolebutyric acid. The concentration of kanamycin was 30 mg·L^−1^.

### 4.11. Cd Content Determination

The RNA extraction and qRT-PCR were conducted as stated above for choosing the three different transgenic lines with similar expression levels (Figures S1 and S2). The WT, *35S::SaARF4* (Figure S1, the red arrows), and *35S::SaARF4-SNPm* (Figure S2, the red arrows) plants were cultured in the same condition (25 °C, 16 h light/8 h dark cycle) for three months. Then, different lines with three biological repeats were cultured under 50 μM CdCl_2_ for 14 d. The Cd content was measured as described before [16]. In detail, the aerial parts of these plants were collected and weighted. Each sample was dried at 60 °C, and treated with concentrated nitric acid at 200 °C. The Cd content was determined by ICP-OES (inductively coupled plasma optical emission spectrometer).

### 4.12. Statistical Analysis

One-way ANOVA was utilized for significance analysis. All data were showed as means ± SD. The least significant difference (LSD) test was applied to analyze differences at a 0.05 or 0.01 level (*, *p* < 0.05; **, *p* < 0.01).

## 5. Conclusions

In this study, bioinformatic analysis was used to screen the candidate gene from the *ARF* family of HE, and then a transgenic method was utilized to identify whether the candidate gene could influence the Cd accumulation of plants. We found that *SaARF4* underwent the positive selection pressure, and there may be a functional divergence between *SaARF4* and other *SaARFs*. Furthermore, *SaARF4* was a hub gene in the coexpression network, and it may affect over 280 genes under Cd stress. The results of qRT-PCR indicated that the expression of *SaARF4* can be induced by Cd stress; therefore, it was chosen as the candidate gene to determine its roles in Cd accumulation. Overexpressing *SaARF4* effectively decreased Cd accumulation of NHE. According to the previous study, the protein structure and surface charge distribution may influence the binding of a transcription factor and the promoter of its downstream genes. We detected an SNP site that can alter the protein structure and charge distribution of *SaARF4*. The technology of site-directed mutagenesis was used to change *SaARF4* sequences based on this SNP site. Overexpressing the mutant of *SaARF4* did not significantly decline the Cd accumulation of NHE. Therefore, we considered that *SaARF4* and its downstream genes may play important roles in Cd accumulation.

## Figures and Tables

**Table 1:** Positive selection sites in each clade.

Clade	Model	npa	lnL	2∆lnL	Positive Selection Sites
I	Model-null	163	−5552.702535		Not allowed
	Model-alte	164	−5552.702535	0	1 M 0.980 *, 9 T 0.984 *, 18 T 0.987 *, 22 I 1.000 **, 30 Q 1.000 **, 36 A 1.000 **, 38 T 1.000 **, 40 E 1.000 **, 42 C 1.000 **, 43 H 0.992 **
II	Model-null	163	−5565.273813		Not allowed
	Model-alte	164	−5565.273813	0	6 M 1.000 **, 23 S 0.971 *
III	Model-null	163	−5570.062526		Not allowed
	Model-alte	164	−5570.062526	0	None
IV	Model-null	163	−5572.824836		Not allowed
	Model-alte	164	−5572.824836	0	None

* means *p* < 0.05 and ** means *p* < 0.01. The reference sequence was *SpARF19*, according to the alignment results.

**Table 2:** Function divergences of *ARF* gene family.

Group 1	Group 2	Type I			Type II
		θI ± s.e.	MFE z-score	LRT	θII ± s.e.
I	II	0.398 ± 0.198	−2.24	5.565 *	−0.581 ± 0.618
I	III	0.270 ± 0.179	−1.65	8.117 **	0.085 ± 0.328
I	IV	0.266 ± 0.167	−1.76	11.987 **	0.194 ± 0.223
II	III	0.215 ± 0.114	−2.20	5.477 *	−0.172 ± 0.505
II	III	0.021 ± 0.074	−0.30	2.723	0.135 ± 0.338
III	IV	0.042 ± 0.070	−0.64	0	0.071 ± 0.258

* means *p* < 0.05 and ** means *p* < 0.01. The reference sequence was *SpARF19*, according to the alignment results.

## Data Availability

Not applicable.

## Supplementary Materials

The following supporting information can be downloaded at: https://www.mdpi.com/article/10.3390/plants11091273/s1, Figure S1: Relative expression level of *SaARF4*; Figure S2: Relative expression level of *SaARF4-SNPm*; Figure S3: SNP sites; Table S1: Primers; Table S2: Basic information; Table S3: Positive selection sites in each clade; Table S4: Function divergences of ARF gene family; Supplementary Material S5: ARFs in Crassulaceae plants; Supplementary Material S6: Cd content.

## References

[1] Tinkov A.A., Filippini T., Ajsuvakova O.P., Skalnaya M.G., Aaseth J., Bjørklund G., Gatiatulina E.R., Popova E.V., Nemereshina O.N., Huang P.-T. (2018). Cadmium and atherosclerosis: A review of toxicological mechanisms and a meta-analysis of epidemiologic studies. Environ. Res..

[2] Rizwan M., Ali S., Adrees M., Ibrahim M., Tsang D.C.W., Zia-ur-Rehman M., Zahir Z.A., Rinklebe J., Tack F.M.G., Ok Y.S. (2017). A critical review on effects, tolerance mechanisms and management of cadmium in vegetables. Chemosphere.

[3] Chaney R.L. (2015). How Does Contamination of Rice Soils with Cd and Zn Cause High Incidence of Human Cd Disease in Sub-sistence Rice Farmers. Curr. Pollut. Rep..

[4] Zhu Y.X., Du W.X., Fang X.Z., Zhang L.L., Jin C.W. (2020). Knockdown of BTS may provide a new strategy to improve cadmi-um-phytoremediation efficiency by improving iron status in plants. J. Hazard. Mater..

[5] Dai F., Luo G., Li Z., Wei X., Wang Z., Lin S., Tang C. (2020). Physiological and transcriptomic analyses of mulberry. Morus atro-purpurea response to cadmium stress. Ecotoxicol. Environ. Saf..

[6] Takahashi R., Ishimaru Y., Shimo H., Ogo Y., Senoura T., Nishizawa N.K., Nakanishi H. (2012). The OsHMA2 transporter is involved in root-to-shoot translocation of Zn and Cd in rice. Plant Cell Environ..

[7] Ueno D., Yamaji N., Kono I., Huang C.F., Ando T., Yano M., Ma J.F. (2010). Gene limiting cadmium accumulation in rice. Proc. Natl. Acad. Sci. USA.

[8] Zhang J., Zhang M., Tian S., Lu L., Shohag M.J.I., Yang X. (2014). Metallothionein 2. SaMT2 from *Sedum alfredii* Hance Confers In-creased Cd Tolerance and Accumulation in Yeast and Tobacco. PLoS ONE.

[9] Guo J., Dai X., Xu W., Ma M. (2008). Overexpressing GSH1 and AsPCS1 simultaneously increases the tolerance and accumulation of cadmium and arsenic in *Arabidopsis thaliana*. Chemosphere.

[10] Shimo H., Ishimaru Y., An G., Yamakawa T., Nakanishi H., Nishizawa N.K. (2011). Low cadmium. LCD, a novel gene related to cadmium tolerance and accumulation in rice. J. Exp. Bot..

[11] Zhao H., Wang L., Zhao F.-J., Wu L., Liu A., Xu W. (2019). SpHMA1 is a chloroplast cadmium exporter protecting photochemical reactions in the Cd hyperaccumulator *Sedum plumbizincicola*. Plant Cell Environ..

[12] Fu S., Lu Y., Zhang X., Yang G., Chao D., Wang Z., Shi M., Chen J., Chao D.-Y., Li R. (2019). The ABC transporter ABCG36 is required for cadmium tolerance in rice. J. Exp. Bot..

[13] Verret F., Gravot A., Auroy P., Leonhardt N., David P., Nussaume L., Vavasseur A., Richaud P. (2004). Overexpression of AtHMA4 enhances root-to-shoot translocation of zinc and cadmium and plant metal tolerance. FEBS Lett..

[14] Wojas S., Hennig J., Plaza S., Geisler M., Siemianowski O., Skłodowska A., Ruszczyńska A., Bulska E., Antosiewicz D.M. (2009). Ectopic expression of Arabidopsis ABC transporter MRP7 modifies cadmium root-to-shoot transport and accumulation. Environ. Pollut..

[15] Russell D., Soulimane T. (2012). Evidence for zinc and cadmium binding in a CDF transporter lacking the cytoplasmic domain. FEBS Lett..

[16] Liu H., Zhao H., Wu L., Liu A., Zhao F.-J., Xu W. (2017). Heavy metal ATPase 3. HMA3 confers cadmium hypertolerance on the cadmium/zinc hyperaccumulator Sedum plumbizincicola. New Phytol..

[17] Pan F., Luo S., Shen J., Wang Q., Ye J., Meng Q., Wu Y., Chen B., Cao X., Yang X. (2017). The effects of endophytic bacterium SaMR12 on *Sedum alfredii* Hance metal ion uptake and the expression of three transporter family genes after cadmium ex-posure. Environ. Sci. Pollut. Res..

[18] Ullah I., Wang Y., Eide D.J., Dunwell J.M. (2018). Evolution, and functional analysis of Natural Resistance-Associated Macrophage Proteins. NRAMPs from Theobroma cacao and their role in cadmium accumulation. Sci. Rep..

[19] Lau S., Jürgens G., De Smet I. (2008). The Evolving Complexity of the Auxin Pathway. Plant Cell.

[20] Zhu X.F., Wang Z.W., Dong F., Lei G.J., Shi Y.Z., Li G.X., Zheng S.J. (2013). Exogenous auxin alleviates cadmium toxicity in *Arabidopsis thaliana* by stimulating synthesis of hemicellulose 1 and increasing the cadmium fixation capacity of root cell walls. J. Hazard. Mater..

[21] Chandler J.W. (2016). Auxin response factors. Plant Cell Environ..

[22] Guilfoyle T.J., Hagen G. (2007). Auxin response factors. Curr. Opin. Plant Biol..

[23] He F., Xu C., Fu X., Shen Y., Guo L., Leng M., Luo K. (2018). The MicroRNA390 TRANS-ACTING SHORT INTERFERING RNA3 Module Mediates Lateral Root Growth under Salt Stress via the Auxin Pathway. Plant Physiol..

[24] Damodharan S., Corem S., Gupta S.K., Arazi T. (2018). Tuning of SlARF10A dosage by sly-miR160a is critical for auxin-mediated compound leaf and flower development. Plant J..

[25] Deng D.M., Shu W.S., Zhang J., Zou H.L., Lin Z., Ye Z.H., Wong M.H. (2007). Zinc and cadmium accumulation and tolerance in populations of *Sedum alfredii*. Environ. Pollut..

[26] Hu Y., Tian S., Foyer C.H., Hou D., Wang H., Zhou W., Liu T., Ge J., Lu L., Lin X. (2019). Efficient phloem transport significantly remobilizes cadmium from old to young organs in a hyperaccumulator *Sedum alfredii*. J. Hazard. Mater..

[27] Deng L., Li Z., Wang J., Liu H., Li N., Wu L., Hu P., Luo Y., Christie P. (2016). Long-term field phytoextraction of zinc/cadmium contaminated soil by Sedum plumbizincicola under different agronomic strategies. Int. J. Phytoremediat..

[28] Jin X., Yang X., Islam E., Liu D., Mahmood Q. (2008). Effects of cadmium on ultrastructure and antioxidative defense system in hyperaccumulator and non-hyperaccumulator ecotypes of *Sedum alfredii* Hance. J. Hazard. Mater..

[29] Yang X.E., Long X.X., Ye H.B., He Z.L., Calvert D.V., Stoffella P.J. (2004). Cadmium tolerance and hyperaccumulation in a new Zn-hyperaccumulating plant species *Sedum alfredii* Hance. Plant Soil.

[30] Li T., Yang X., Lu L., Islam E., He Z. (2009). Effects of zinc and cadmium interactions on root morphology and metal translocation in a hyperaccumulating species under hydroponic conditions. J. Hazard. Mater..

[31] Liu J., Chen N., Chen F., Cai B., Dal Santo S., Tornielli G.B., Pezzotti M., Cheng Z.-M. (2014). Genome-wide analysis and expression profile of the bZIP transcription factor gene family in grapevine (*Vitis vinifera*). BMC Genom..

[32] Cannon S.B., Mitra A., Baumgarten A., Young N.D., May G. (2004). The roles of segmental and tandem gene duplication in the evolution of large gene families in *Arabidopsis thaliana*. BMC Plant Biol..

[33] Abrouk M., Murat F., Pont C., Messing J., Jackson S., Faraut T., Tannier E., Plomion C., Cooke R., Feuillet C. (2010). Palaeogenomics of plants: Synteny-based modelling of extinct ancestors. Trends Plant Sci..

[34] Han X., Yin H., Song X., Zhang Y., Liu M., Sang J., Jiang J., Li J., Zhuo R. (2016). Integration of small RNAs, degradome and transcriptome sequencing in hyperaccumulator *Sedum alfredii* uncovers a complex regulatory network and provides insights into cadmium phytoremediation. Plant Biotechnol. J..

[35] Wang B., Li M., Yuan Y., Liu S. (2019). Genome-Wide Comprehensive Analysis of the SABATH Gene Family in Arabidopsis and Rice. Evol. Bioinform..

[36] Maniatis T., Reed R. (2002). An extensive network of coupling among gene expression machines. Nature.

[37] Liu H., Lyu H.-M., Zhu K., Van de Peer Y., Cheng Z.-M. (2021). The emergence and evolution of intron-poor and intronless genes in intron-rich plant gene families. Plant J..

[38] Guilfoyle T.J. (2015). The PB1 Domain in Auxin Response Factor and Aux/IAA Proteins: A Versatile Protein Interaction Module in the Auxin Response. Plant Cell.

[39] Zhang J., Wu A., Wei H., Hao P., Zhang Q., Tian M., Yang X., Cheng S., Fu X., Ma L. (2020). Genome-wide identification and expression patterns analysis of the RPD3/HDA1 gene family in cotton. BMC Genom..

[40] Kong Y., Xu P., Jing X., Chen L., Li L., Li X. (2017). Decipher the ancestry of the plant-specific LBD gene family. BMC Genom..

[41] Marin E., Jouannet V., Herz A., Lokerse A.S., Weijers D., Vaucheret H., Nussaume L., Crespi M.D., Maizel A. (2010). miR390, Arabidopsis TAS3 tasiRNAs, and Their AUXIN RESPONSE FACTOR Targets Define an Autoregulatory Network Quantitatively Regulating Lateral Root Growth. Plant Cell.

[42] Liu K., Yuan C., Li H., Lin W., Yang Y., Shen C., Zheng X. (2015). Genome-wide identification and characterization of auxin response factor (ARF) family genes related to flower and fruit development in papaya (*Carica papaya* L.). BMC Genom..

[43] Xu C., Shen Y., He F., Fu X., Yu H., Lu W., Li Y., Li C., Fan D., Wang H.C. (2019). Auxin-mediated Aux/IAA-ARF-HB signaling cascade regulates secondary xylem development in Populus. New Phytol..

[44] Okushima Y., Overvoorde P.J., Arima K., Alonso J.M., Chan A., Chang C., Ecker J.R., Hughes B., Lui A., Nguyen D. (2005). Functional Genomic Analysis of the AUXIN RESPONSE FACTOR Gene Family Members in *Arabidopsis thaliana*: Unique and Overlapping Functions of ARF7 and ARF19. Plant Cell.

[45] Ding B., Xia R., Lin Q., Gurung V., Sagawa J.M., Stanley L.E., Strobel M., Diggle P.K., Meyers B.C., Yuan Y.-W. (2020). Developmental Genetics of Corolla Tube Formation: Role of the tasiRNA-ARF Pathway and a Conceptual Model. Plant Cell.

[46] Yin H., Li M., Lv M., Hepworth S.R., Li D., Ma C., Li J., Wang S.-M. (2020). SAUR15 Promotes Lateral and Adventitious Root Development via Activating H^+^-ATPases and Auxin Biosynthesis. Plant Physiol..

[47] Banerjee-Basu S., Baxevanis A.D. (2004). Structural analysis of disease-causing mutations in the P-subfamily of forkhead transcription factors. Proteins Struct. Funct. Bioinform..

[48] Shanahan H.P., Garcia M.A., Jones S., Thornton J.M. (2004). Identifying DNA-binding proteins using structural motifs and the electrostatic potential. Nucleic Acids Res..

[49] Ofran Y., Mysore V., Rost B. (2007). Prediction of DNA-binding residues from sequence. Bioinformatics.

[50] Shuang-Shuang C., Jing J., Xiao-Jiao H., Yun-Xing Z., Ren-Ying Z. (2018). Identification, Expression Analysis of the Hsf Family, and Characterization of Class A4 in *Sedum alfredii* Hance under Cadmium Stress. Int. J. Mol. Sci..

[51] Goodstein D.M., Shu S., Howson R., Neupane R., Hayes R.D., Fazo J., Mitros T., Dirks W., Hellsten U., Putnam N. (2012). Phytozome: A comparative platform for green plant genomics. Nucleic Acids Res..

[52] Xu D., Lu Z., Jin K., Qiu W., Qiao G., Han X., Zhuo R. (2021). SPDE: A Multi-functional Software for Sequence Processing and Data Extraction. Bioinformatics.

[53] Chen C., Chen H., He Y., Xia R. (2018). TBtools, a Toolkit for Biologists integrating various biological data handling tools with a user-friendly interface. BioRxiv.

[54] Smoot M.E., Ono K., Ruscheinski J., Wang P.-L., Ideker T. (2011). Cytoscape 2.8: New features for data integration and network visualization. Bioinformatics.

[55] Gu X., Zou Y., Su Z., Huang W., Zhou Z., Arendsee Z., Zeng Y. (2013). An Update of DIVERGE Software for Functional Divergence Analysis of Protein Family. Mol. Biol. Evol..

[56] Liu N., Dong L., Deng X., Liu D., Liu Y., Li M., Hu Y., Yan Y. (2018). Genome-wide identification, molecular evolution, and expression analysis of auxin response factor (ARF) gene family in *Brachypodium distachyon* L.. BMC Plant Biol..

[57] Bradbury P.J., Zhang Z., Kroon D.E., Casstevens T.M., Ramdoss Y., Buckler E.S. (2007). TASSEL: Software for association mapping of complex traits in diverse samples. Bioinformatics.

[58] Pettersen E.F., Goddard T.D., Huang C.C., Couch G.S., Greenblatt D.M., Meng E.C., Ferrin T.E. (2004). UCSF Chimera—A visualization system for exploratory research and analysis. J. Comput. Chem..

